# Risk factor analysis for early rebleeding after endoscopic treatment for colonic diverticular bleeding with stigmata of recent hemorrhage

**DOI:** 10.1002/jgh3.12535

**Published:** 2021-03-27

**Authors:** Atsushi Yamauchi, Tadayuki Kou, Takuya Kishimoto, Yuki Mori, Kazuki Osawa, Kei Iimori, Kosuke Iwano, Yuya Kawai, Kenji Sawada, Kensuke Hamada, Satoshi Nishimura, Yoshiharu Mori, Kotaro Watanabe, Shunjiro Azuma, Toshihiro Morita, Akira Kurita, Kiyotaka Kawaguchi, Yoshiki Suginoshita, Toshiro Katayama, Shujiro Yazumi

**Affiliations:** ^1^ Department of Gastroenterology and Hepatology Tazuke Kofukai Medical Research Institute, Kitano Hospital Osaka Japan; ^2^ Department of Medical Engineering, Faculty of Health Science Morinomiya University of Medical Sciences Osaka Japan

**Keywords:** endoscopic band ligation, endoscopic clipping, endoscopic modality, left‐sided colon, lower gastrointestinal bleeding

## Abstract

**Background and Aim:**

Colonic diverticular bleeding is a common cause of acute lower gastrointestinal bleeding. Endoscopic hemostasis is generally selected as the first‐line treatment; however, a considerable number of patients experience early rebleeding after endoscopic treatment. We investigated the risk factors for early rebleeding after endoscopic treatment.

**Methods:**

We retrospectively evaluated the data of 142 consecutive patients who underwent endoscopic treatment (endoscopic clipping or endoscopic band ligation) for colonic diverticular bleeding with stigmata of recent hemorrhage between April 2012 and April 2020. Multivariate logistic regression analysis was conducted to evaluate the statistical relationship between patient characteristics and the incidence of early rebleeding occurring within 30 days after endoscopic treatment.

**Results:**

Of 142 patients, early rebleeding was detected in 34 (23.9%) patients. According to univariate analysis, platelet count of <10 × 10^4^/μL, bleeding from the left‐sided colon, and endoscopic clipping usage were associated with early rebleeding (*P* < 0.05). The subsequent multivariate logistic regression analysis identified bleeding from the left‐sided colon (odds ratio [OR], 4.16; 95% confidence interval [CI], 1.73–10.0; *P* = 0.001) and endoscopic clipping usage (OR, 2.92; 95% CI, 1.21–7.00; *P* = 0.017) as the independent risk factors for early rebleeding.

**Conclusions:**

Bleeding from the left‐sided colon and endoscopic clipping usage were the risk factors for early rebleeding after endoscopic treatment. Using endoscopic band ligation was associated with a decreased risk for early rebleeding compared with the use of endoscopic clipping, indicating that endoscopic band ligation was a preferable endoscopic modality to prevent early recurrent bleeding.

## Graphical abstract

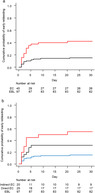

## Introduction

Colonic diverticular bleeding is the common cause of acute lower gastrointestinal bleeding worldwide.^1^ In the majority of cases, it spontaneously ameliorates; however, in cases where spontaneous hemostasis is not acquired, endoscopic hemostasis is generally selected as the first‐line treatment.^2^

A variety of endoscopic modalities are now clinically available for the endoscopic treatment of colonic diverticular bleeding.^3^, ^4^, ^5^, ^6^ However, conventional endoscopic modalities such as epinephrine injection, contact coagulation, and endoscopic clipping (EC) are generally associated with high early bleeding rates, and patients often experience recurrent bleeding immediately after endoscopic treatment.^2^, ^3^, ^6^ In this situation, endoscopic band ligation (EBL), which was first introduced by Farrell *et al*. in 2003, is emerging as a promising endoscopic modality for treating colonic diverticular bleeding, and its efficacy and safety have been demonstrated by several clinical studies.^7^, ^8^, ^9^, ^10^, ^11^, ^12^, ^13^

This study aimed to explore the risk factors for early rebleeding in patients undergoing endoscopic treatment for colonic diverticular bleeding with stigmata of recent hemorrhage (SRH).

## Methods

### Study design

We evaluated the data of 142 consecutive patients who underwent endoscopic treatment (EC or EBL) for acute colonic diverticular bleeding between April 2012 and April 2020 at Kitano Hospital. All patients were endoscopically diagnosed as having definite colonic diverticular bleeding based on the presence of SRH such as active bleeding from a diverticulum, a nonbleeding visible vessel within a diverticulum, or a densely adherent clot despite vigorous irrigation. Early rebleeding was defined as bleeding occurring within 30 days after endoscopic treatment with clinical evidence of recurrent lower gastrointestinal bleeding, such as hemorrhagic shock.^14^

This study was conducted according to the principles of the Declaration of Helsinki and was reviewed and approved by the institutional review board at Kitano Hospital. EBL was performed under clinical study (UMIN000021316) until June 2018 when it was approved as an endoscopic modality for treating colonic diverticular bleeding in Japan.

### Endoscopic treatment

All patients underwent a colonoscopy with a water‐jet scope without sedation within 24 h from arrival at our hospital. Bowel preparation was performed using polyethylene glycol solution or enema. A transparent soft hood was attached to the tip of the scope to identify the responsible diverticulum.

Endoscopic hemostasis was performed with EC or EBL. EC was performed using hemoclips (Olympus Medical Systems Co, Ltd., Tokyo, Japan) by placing them directly on a responsible vessel within a diverticulum (direct EC method) or by closing an orifice of the responsible diverticulum (indirect EC method).^2^ In this study, we retrospectively classified the patients treated with EC into direct and indirect EC groups using the endoscopic reports. Regarding EBL, a marker clip was first placed near the responsible diverticulum, and the colonoscope was removed and reinserted after attaching the EBL device (Sumitomo Bakelite Co, Ltd., Tokyo, Japan) at the tip of the colonoscope. The responsible diverticulum was suctioned into the EBL device, and the elastic O‐band was released. The endoscopic modality was selected at the discretion of the treating endoscopist.

### Statistical analysis

A logistic regression model was used to estimate the odds ratio (OR). The time‐to‐event analysis was performed using the Kaplan–Meier method and the log‐rank test. *P* values <0.05 were considered statistically significant. Statistical analysis was performed using EZR (Saitama Medical Center, Jichi Medical University, Saitama, Japan).^15^

## Results

### Patient characteristics

Table 1 shows the baseline characteristics of the patients. The median age of the patients was 75 years (range, 30–93 years). Of the 142 patients, 45 (31.7%) were treated with EC, and the remaining 97 patients (68.3%) were treated with EBL. Among the 45 patients treated with EC, direct EC was performed in 20 patients, and indirect EC was performed in 25 patients. Hypertension was the most common comorbidity (77.5%). Bowel preparation was performed in 105 patients (73.9%). More than half of the responsible diverticulum was located at the right‐sided colon (73.9%).

**Table 1:** Characteristics of patients who underwent endoscopic treatment for colonic diverticular bleeding with stigmata of recent hemorrhage

Characteristics	EC (%) (*n* = 45)	EBL (%) (*n* = 97)	Total (%) (*n* = 142)
Age			
**<**80	25 (55.6)	69 (71.1)	94 (66.2)
≥80	20 (44.4)	28 (28.9)	48 (33.8)
Sex			
Male	36 (80.0)	82 (84.5)	118 (83.1)
Female	9 (20.0)	15 (15.5)	24 (16.9)
BMI (kg/m^2^)			
**<**25.0	32 (71.1)	69 (71.1)	101 (71.1)
≥25.0	13 (28.9)	28 (28.9)	41 (28.9)
Systolic blood pressure (mmHg)			
**<**90	2 (4.4)	3 (3.1)	5 (3.5)
≥90	43 (95.6)	94 (96.9)	137 (96.5)
Heart rate (bpm)			
**<**100	37 (82.2)	73 (75.3)	110 (77.5)
≥100	8 (17.8)	24 (24.7)	32 (22.5)
Blood transfusion	23 (51.1)	32 (33.0)	55 (38.7)
Medication			
Antiplatelet agents	15 (33.3)	39 (40.2)	54 (38.0)
Anticoagulants	10 (22.2)	16 (16.5)	26 (18.3)
NSAIDs	4 (8.9)	10 (10.3)	14 (9.9)
Comorbidity			
Hypertension	34 (75.6)	76 (78.4)	110 (77.5)
Hyperlipidemia	16 (35.6)	36 (37.1)	52 (36.6)
Diabetes mellitus	10 (22.2)	25 (25.8)	35 (24.6)
Maintenance dialysis	5 (11.1)	5 (5.2)	10 (7.0)
Ischemic heart disease	10 (22.2)	13 (13.4)	23 (16.2)
Cerebrovascular disease	9 (20.0)	25 (25.8)	34 (23.9)
Hemoglobin (g/dL)			
**<**8.0	10 (22.2)	25 (25.8)	35 (24.6)
≥8.0	35 (77.8)	72 (74.2)	107 (75.4)
Platelet count (×10^4^/μL)			
**<**10.0	7 (15.6)	2 (2.1)	9 (6.3)
≥10.0	38 (84.4)	95 (97.9)	133 (93.7)
Albumin (mg/dL)			
**<**3.0	5 (11.1)	13 (13.4)	18 (12.7)
≥3.0	40 (88.9)	84 (86.6)	124 (87.3)
Creatinine (mg/dL)			
**<**1.0	28 (62.2)	67 (69.1)	95 (66.9)
≥1.0	17 (37.8)	30 (30.9)	47 (33.1)
Radiology			
Urgent CECT	32 (71.1)	78 (80.4)	110 (77.5)
Extravasation on CECT	18 (40.0)	32 (33.0)	50 (35.2)
Bowel preparation	27 (60.0)	78 (80.4)	105 (73.9)
Enema	4 (8.9)	22 (22.7)	26 (18.3)
PEG	23 (51.1)	56 (57.7)	79 (55.6)
Location of bleeding			
Right‐sided colon	28 (62.2)	77 (79.4)	105 (73.9)
Left‐sided colon	17 (37.8)	20 (20.6)	37 (26.1)
SRH			
Active bleeding	23 (51.1)	63 (64.9)	86 (60.6)
Nonbleeding visible vessel	14 (31.1)	21 (21.6)	35 (24.6)
Adherent clot	8 (17.8)	13 (13.4)	21 (14.8)

Abbreviations: BMI, body mass index; CECT, contrast‐enhanced computed tomography; EBL, endoscopic band ligation; EC, endoscopic clipping; NSAIDs, nonsteroidal anti‐inflammatory drugs; PEG, polyethylene glycol; SRH, stigmata of recent hemorrhage.

### Incidence of early rebleeding after endoscopic treatment

Of the 142 patients, 34 (23.9%) experienced early rebleeding within 30 days after endoscopic treatment. The median duration from endoscopic treatment to rebleeding was 2 days (range, 1–21 days). Table 2 shows the rates of early rebleeding according to each of the patient characteristics.

**Table 2:** Univariate analysis of risk factors for early rebleeding

Characteristics	Number of patients	Early rebleeding rate (%)	OR (95% CI)	*P* value
Age				
**<**80	94	23/94 (24.5)	1	
≥80	48	11/48 (22.9)	0.92 (0.40–2.09)	0.838
Sex				
Male	118	27/118 (22.9)	1	
Female	24	7/24 (29.2)	1.39 (0.52–3.70)	0.512
BMI (kg/m^2^)				
**<**25.0	101	22/101 (21.8)	1	
≥25.0	41	12/41 (29.3)	1.49 (0.65–3.38)	0.345
Systolic blood pressure (mmHg)				
**<**90	5	1/5 (20.0)	1	
≥90	137	33/137 (24.1)	1.27 (0.14–11.8)	0.834
Heart rate (bpm)				
**<**100	110	27/110 (24.5)	1	
≥100	32	7/32 (21.9)	0.86 (0.34–2.21)	0.756
Blood transfusion	55	16/55 (29.1)	1.57 (0.72–3.43)	0.255
Medication				
Antiplatelet agents	54	16/54 (29.6)	1.64 (0.75–3.57)	0.216
Anticoagulants	26	5/26 (19.2)	0.71 (0.25–2.07)	0.535
NSAIDs	14	6/14 (42.9)	2.68 (0.86–8.36)	0.090
Comorbidity				
Hypertension	110	26/110 (23.6)	0.93 (0.37–2.31)	0.874
Hyperlipidemia	52	16/52 (30.8)	1.78 (0.81–3.89)	0.150
Diabetes mellitus	35	7/35 (20.0)	0.74 (0.29–1.89)	0.530
Maintenance dialysis	10	3/10 (30.0)	1.40 (0.34–5.73)	0.643
Ischemic heart disease	23	6/23 (26.1)	1.15 (0.41–3.19)	0.793
Cerebrovascular disease	34	6/34 (17.6)	0.61 (0.23–1.63)	0.327
Hemoglobin (g/dL)				
**<**8.0	35	8/35 (22.9)	1	
≥8.0	107	26/107 (24.3)	1.08 (0.44–2.68)	0.862
Platelet count (×10^4^/μL)				
≥10.0	133	29/133 (21.8)	1	
**<**10.0	9	5/9 (55.6)	4.48 (1.13–17.80)	0.033
Albumin (mg/dL)				
**<**3.0	18	3/18 (16.7)	1	
≥3.0	124	31/124 (25.0)	1.67 (0.45–6.14)	0.443
Creatinine (mg/dL)				
**<**1.0	95	22/95 (23.2)	1	
≥1.0	47	12/47 (25.5)	1.14 (0.51–2.56)	0.755
Radiology				
Urgent CECT	110	28/110 (25.5)	1.48 (0.55–3.97)	0.436
Extravasation on CECT	50	13/50 (26.0)	1.19 (0.54–2.64)	0.672
Bowel preparation	105	21/105 (20.0)	0.46 (0.20–1.06)	0.067
Enema	26	5/26 (19.2)	0.71 (0.25–2.07)	0.535
PEG	79	16/79 (20.3)	0.64 (0.29–1.38)	0.250
Location of bleeding				
Right‐sided colon	105	17/105 (16.2)	1	
Left‐sided colon	37	17/37 (45.9)	4.4 (1.92–10.1)	0.0004
SRH				
Active bleeding	86	23/86 (26.7)	1.49 (0.66–3.37)	0.334
Nonbleeding visible vessel	35	5/35 (14.3)	0.45 (0.16–1.27)	0.130
Adherent clot	21	6/21 (28.6)	1.33 (0.47–3.75)	0.591
Endoscopic modality				
EBL	97	15/97 (15.5)	1	
EC	45	19/45 (42.2)	3.99 (1.78–8.96)	0.0007

Abbreviations: BMI, body mass index; CECT, contrast‐enhanced computed tomography; CI, confidence interval; EBL, endoscopic band ligation; EC, endoscopic clipping; NSAIDs, nonsteroidal anti‐inflammatory drugs; OR, odds ratio; PEG, polyethylene glycol; SRH, stigmata of recent hemorrhage.

### Identification of risk factors for early rebleeding by multivariate analysis

Tables 2 and 3 summarize the results of univariate and multivariate analyses, respectively. Among 29 factors tested in the univariate analysis, the following three factors were identified as significant (*P* < 0.05): platelet count **<**10 × 10^4^/μL, bleeding from the left‐sided colon, and the use of EC. After multivariate logistic regression analysis, the following two factors remained as independent risk factors: bleeding from the left‐sided colon (OR, 4.16; 95% confidence interval [CI], 1.73–10.0; *P* = 0.001) and the use of EC (OR, 2.92; 95% CI, 1.21–7.00; *P* = 0.017).

**Table 3:** Multivariate analysis of risk factors for early rebleeding

Characteristics	Number of patients	OR (95% CI)	*P* value
Platelet count			
>10.0 × 10^4^/μL	133	1	
≤10.0 × 10^4^/μL	9	3.50 (0.79–15.5)	0.099
Location of bleeding			
Right‐sided colon	105	1	
Left‐sided colon	37	4.16 (1.73–10.0)	0.001
Endoscopic modality			
EBL	97	1	
EC	45	2.92 (1.21–7.00)	0.017

Abbreviations: CI, confidence interval; EC, endoscopic clipping; EBL, endoscopic band ligation; OR, odds ratio.

### Time‐to‐event analysis for early rebleeding after endoscopic treatment

**Figure 1:**
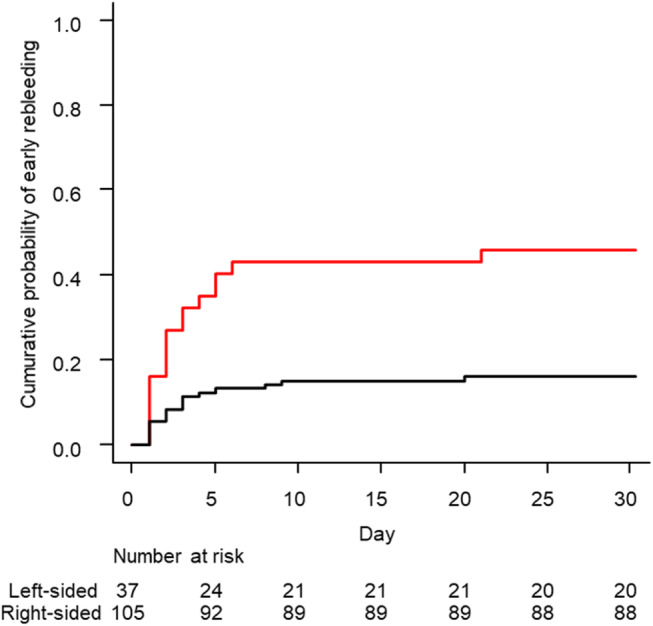
Early rebleeding after endoscopic treatment for the right‐ or left‐sided colonic diverticular bleeding with stigmata of recent hemorrhage. Cumulative probability of recurrent bleeding in the right‐ and left‐sided colon groups. A significant difference was observed in the early rebleeding rate (*P* < 0.05). 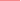, Left‐sided; 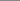, right‐sided.

**Figure 2:**
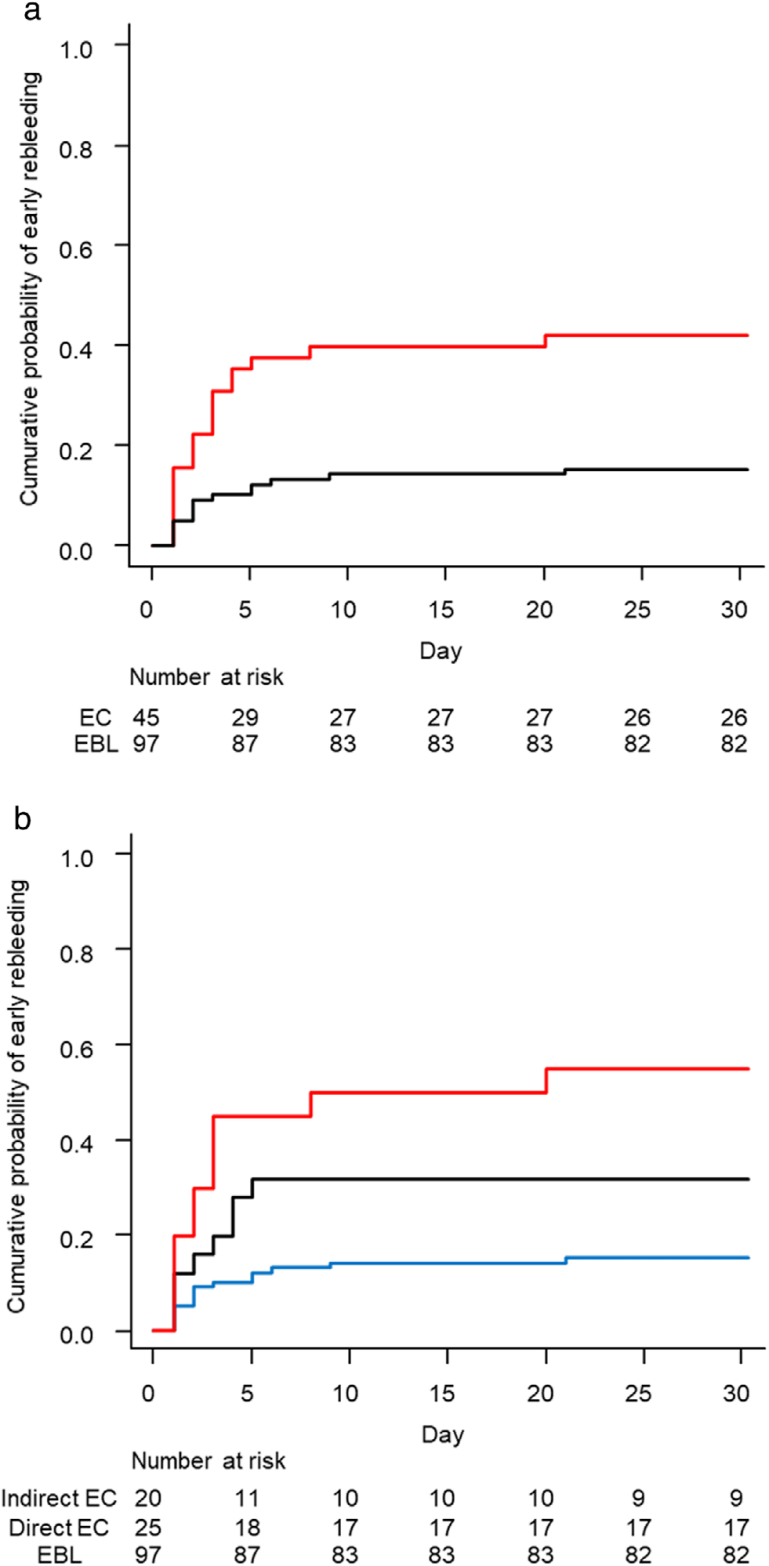
Early rebleeding after endoscopic band ligation (EBL) or endoscopic clipping (EC) for colonic diverticular bleeding with stigmata of recent hemorrhage. (a) Cumulative probability of recurrent bleeding in the EBL and EC groups. 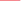, EC; 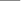, EBL. (b) Cumulative probability of recurrent bleeding in the EBL, and direct and indirect EC groups. A significant difference was observed in the early rebleeding rate (*P* < 0.05). 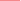, Indirect EC; 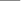, direct EC; 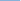, EBL.

Figures 1 and 2 show the results of the time‐to‐event analysis for early bleeding performed using the Kaplan–Meier method. First, regarding the location of diverticular bleeding, the cumulative probabilities of early rebleeding at 5, 10, and 15 days were 35.1%, 43.2%, and 43.2% in the left‐sided colon group and 12.4%, 15.2%, and 15.2% in the right‐sided colon group, respectively. The cumulative probability of early rebleeding at 30 days was estimated to be significantly lower with bleeding from the right‐sided colon at 16.2% (95% CI, 10.4–24.7%) than with bleeding from the left‐sided colon at 45.9% (95% CI, 31.6–63.1%, *P* < 0.05; Fig. 1).

Next, regarding endoscopic modality, the cumulative probabilities of early rebleeding at 5, 10, and 15 days were 35.6%, 40.0%, and 40.0% in the EC group and 10.3%, 14.4%, and 14.4% in the EBL group, respectively. The cumulative probability of early rebleeding at 30 days was estimated to be significantly lower with the use of EBL at 15.5% (95% CI, 9.6–24.3%) than with the use of EC at 42.2% (95% CI, 29.4–57.9%, *P* < 0.05; Fig. 2a). We also classified the patients treated with EC into direct and indirect EC groups to compare the data between both groups. Consequently, the cumulative probability of early rebleeding at 30 days was estimated to be significantly lower with the use of EBL at 15.5% (95% CI, 9.6–24.3%) than not only with the use of indirect EC at 55.0% (95% CI, 35.3–76.9%, *P* < 0.05) but also with the use of direct EC at 32.0% (95% CI, 17.5–53.9%, *P* < 0.05; Fig. 2b).

## Discussion

In this study, we observed that 34 of 142 patients (23.9%) experienced early rebleeding within 30 days after endoscopic treatment, and bleeding from the left‐sided colon and the use of EC were found to be significantly associated with an increased risk for early rebleeding. Conversely, the patient characteristics such as age, anticoagulant intake, and comorbidity were not associated with an increased risk.

EC is widely used as an endoscopic modality for treating colonic diverticular bleeding because of its simple operability in daily clinical practice; however, it is generally associated with high early rebleeding rates, ranging from 0 to 50%.^3^ Several possible reasons could be attributed to the high rebleeding rate associated with EC use. Placing a clip directly on a responsible vessel within a diverticulum is required to acquire an effective hemostasis with EC; however, in a majority of cases of acute colonic diverticular bleeding, it is difficult to identify the responsible vessel because of massive bleeding. Moreover, even when the responsible vessel can be identified, the orifice of the diverticulum is generally small, making it difficult to place a clip directly on the responsible vessel. By contrast, EBL can tightly ligate the inverted diverticulum with the responsible vessel, thus achieving effective hemostasis even in patients with massive bleeding.

Regarding the comparison of efficacy between EC and EBL for colonic diverticular bleeding, to our knowledge, four pivotal previous studies have investigated the early rebleeding rate (Table 4).^9^, ^16^, ^17^, ^18^ Consistent with our study, three studies have reported that EBL is superior to EC with regard to preventing early rebleeding. For EC, Kishino *et al*. recently reported that the early bleeding rate of direct EC was comparable with that of EBL and that direct EC could be an alternative as the first‐line treatment.^19^ However, in the present study, the early bleeding rate of EBL was significantly lower than that of both indirect and direct EC, suggesting the superiority of EBL to EC as an endoscopic modality for treating colonic diverticular bleeding with regard to preventing early rebleeding.

**Table 4:** Previous and present studies investigating the early rebleeding rate in patients treated with EBL or EC for colonic diverticular bleeding

	Number of patients	Early rebleeding rate (%)		OR (95% CI)	*P* value
		EBL	EC		
Setoyama *et al*.^9^	66	5.6	33.3	—	0.018*
Nakano *et al*.^16^	100	14.0	38.0	—	0.004**
Nagata *et al*.^17^	108	9.8	21.3	—	0.097*
Honda *et al*.^18^	141	8.7	36.8	—	0.0002*
Present study	142	15.5	42.2	2.92 (1.21–7.00)	0.017***

* Chi‐square test.

** Log‐rank test.

*** Logistic regression analysis.

Abbreviations: CI, confidence interval; EBL, endoscopic band ligation; EC, endoscopic clipping; OR, odds ratio.

In the present study, bleeding from the left‐sided colon was identified as an independent risk factor for early rebleeding after endoscopic treatment. Consistent with our result, several studies have reported that bleeding from the left‐sided colon is associated with an increased risk for recurrent bleeding after endoscopic treatment.^12^, ^18^ However, some other studies have reported that bleeding from the right‐sided colon has a higher risk of recurrent bleeding.^20^, ^21^ Therefore, whether the location of bleeding can influence the clinical outcome after endoscopic treatment is unclear. However, we speculate that the higher rebleeding rate in the left‐sided colon could be attributed to the difficulty of endoscopic treatment due to lower maneuverability of the endoscope in the left‐sided, especially sigmoid, colon than in the right‐sided colon. Moreover, the wall of the left‐sided colon is supposed to be anatomically thicker than that of the right‐sided colon.^22^ Therefore, regarding EBL, the wall of the left‐sided colon is not only hard to be suctioned into the EBL device but there is also a possibility that the elastic O‐band may come off easily in the left‐sided colon.

Our study had some limitations. First, the risk factors were derived from retrospective data at a single institution, and the sample size was limited, due to which the findings could be susceptible to a bias in data collection. Second, although endoscopic treatments were conducted or supervised by expert endoscopists, technical skills may have differed among each endoscopist. Thus, such difference might have affected the clinical outcome of patients after endoscopic treatment. Third, although we evaluated the efficacy of EBL and EC based on short‐term recurrent bleeding risk, the efficacy based on long‐term recurrent bleeding risk should also be verified. Despite these limitations, our study is a large retrospective cohort analysis of the risk factors for early rebleeding in patients undergoing endoscopic treatment for colonic diverticular bleeding with SRH. As shown in Table 4, four previous studies have reported the early rebleeding rate in patients treated with EBL or EC for colonic diverticular bleeding. However, the sample sizes of those studies might not have been sufficient. In this study, we evaluated the efficacies of EBL and EC on the basis of short‐term recurrent bleeding risk in a larger cohort.

In conclusion, bleeding from the left‐sided colon and the use of EC were the independent risk factors for early rebleeding after endoscopic treatment for colonic diverticular bleeding with SRH. Especially, the use of EBL was significantly associated with a decreased risk for early rebleeding compared with that with EC, and our findings indicate that EBL is a preferable endoscopic modality for preventing early recurrent bleeding.
